# Neuropeptides Exert Direct Effects on Rat Thymic Epithelial Cells in Culture

**DOI:** 10.1155/1998/41349

**Published:** 1998

**Authors:** Gail M. Head, R. Mentlein, Birte Von Patay, J. E.G. Downing, Marion D. Kendall

**Affiliations:** 1 Department of Biology Imperial College London SW7 2BB UK; 2 Anatomisches Institute Kiel Germany; 3 Thymus Laboratory The Babraham Institute Cambridge CB2 4AT UK

**Keywords:** Thymic epithelial cells, gap junctions, proliferation, neuropeptides, thymulin, multinucleate cells

## Abstract

To determine if major thymic neuropeptides and neurotransmitters can directly influence the functional activity of cultured rat thymic epithelium, neuropeptides and neurotransmitters were applied, and intercellular communication, proliferation, and thymulin secretion assessed. After injections of a mixture of lucifer yellow dextran (too large to pass gap junctions) and cascade blue (which does) into single cells, some neuropeptides decrease dye coupling: 0.1 mM GABA (*P* < 0.0001), 100 nM NPY (*P* < 0.0001), 100 nM VIP (*P* < 0.001), 100 nM CGRP (*P* < 0.001), 100 nM SP (*P* < 0.01), and 0.1 mM histamine (*P* < 0.01), whereas 0.1 mM 5-HT, mM acetylcholine, and 1 _μ_M isoproterenol (β-adrenergic agonist) had no effect. Proliferation (incorporation of tritiated thymidine) was increased by CGRP (*P* = 0.004) and histamine (*P* < 0.02), but decreased by isoproterenol (*P* = 0.002), 5-HT (*P* = 0.003), and acetylcholine (*P* < 0.05). The percentage of multinucleate cells was decreased after isoproterenol (2.5%), and increased after 5-HT (21.3%), GABA (15%), and histamine (15.1%). Compared to controls, thymulin in the supernatant was decreased after challenge with acetylcholine (52%), isoproterenol (71%), 5-HT (73%), and histamine (84%). This study demonstrates direct effects of neuropeptides and neurotransmitters on functional aspects of cultured thymic epithelial cells.


*Developmental Immunology*, 1998, Vol. 6, pp. 95–104
Reprints available directly from the publisher
Photocopying permitted by license only

© 1998 OPA (Overseas Publishers Association)
N.V. Published by license under the
Harwood Academic Publishers imprint,
part of the Gordon and Breach Publishing Group
Printed in Malaysia

### Neuropeptides Exert Direct Effects on Rat Thymic Epithelial Cells in Culture

GAIL M. HEAD[a], R. MENTLEIN[b], BIRTE VON PATAY[b], J.E.G. DOWNING[a] and MARION D. KENDALL[c*]

[a]*Department of Biology, Imperial College, London SW7 2BB*; [b]*Anatomisches Institute, Kiel, Germany*; [c]*Thymus Laboratory, The Babraham Institute, Cambridge, CB2 4AT*

*(In final form 30 May 1997)*

To determine if major thymic neuropeptides and neurotransmitters can directly influence the functional activity of cultured rat thymic epithelium, neuropeptides and neurotransmitters were applied, and intercellular communication, proliferation, and thymulin secretion assessed. After injections of a mixture of lucifer yellow dextran (too large to pass gap junctions) and cascade blue (which does) into single cells, some neuropeptides decrease dye coupling: 0.1 mM GABA ($P < 0.0001$), 100 nM NPY ($P < 0.0001$), 100 nM VIP ($P < 0.001$), 100 nM CGRP ($P < 0.001$), 100 nM SP ($P < 0.01$), and 0.1 mM histamine ($P < 0.01$), whereas 0.1 mM 5-HT, 1 mM acetylcholine, and 1 $\mu$M isoproterenol ($\beta$-adrenergic agonist) had no effect. Proliferation (incorporation of tritiated thymidine) was increased by CGRP ($P = 0.004$) and histamine ($P < 0.02$), but decreased by isoproterenol ($P = 0.002$), 5-HT ($P = 0.003$), and acetylcholine ($P < 0.05$). The percentage of multinucleate cells was decreased after isoproterenol (2.5%), and increased after 5-HT (21.3%), GABA (15%), and histamine (15.1%). Compared to controls, thymulin in the supernatant was decreased after challenge with acetylcholine (52%), isoproterenol (71%), 5-HT (73%), and histamine (84%). This study demonstrates direct effects of neuropeptides and neurotransmitters on functional aspects of cultured thymic epithelial cells.

*Keywords:* Thymic epithelial cells, gap junctions, proliferation, neuropeptides, thymulin, multinucleate cells

### INTRODUCTION

Many cells commonly communicate with each other through gap junctions. The electrical and metabolic coupling between cells creates functional syncytia and coordinates responses. In smooth or cardiac muscle, electrical coupling through gap junctions synchronizes contraction. Gap junctions also allow the passage of small water-soluble molecules of <15 kD (e.g., amino acids, second messengers, sugars, and inorganic ions) (Loewenstein, 1979). The presence of gap junctions inhibits proliferation and promotes terminal differentiation (Trosko et al., 1993), although the mechanisms vary in different cells. Secretory

*Corresponding author.

activities also can be modulated and initiated, usually through transmission of calcium signals (Meda et al., 1991; Pandol, 1994). Regulation of cellular activities by gap junctions is complex because the regulation of gap-junction permeability is not an "all or nothing" phenomenon. Reduced junctional permeability allows differential movement of modulators. Changing gap-junction permeability also can regulate frequency responses of signal transmission within the syncytia (Santos-Sacchi, 1991; Ngezhayo and Kolb, 1993).

The genes encoding the constituent proteins of these channels are highly conserved. Each protein (connexin) has four transmembrane domains and forms one subunit of the hexameric connexon (Kumar and Gilula, 1992; Yeager and Nicholson, 1996). The connexons are inserted into the membrane and form a complete gap junction when they are aligned with corresponding units on the adjacent cell. Intercellular permeability can be regulated by the rapid assembly and disassembly of gap junctions, or by modulation of the gap-junction pore permeability. The ensemble of intracellular signals, such as alterations to internal pH, temperature, internal calcium, cyclic nucleotides, and the activity of tyrosine and serine kinases, combines to effect changes. In addition, the available connexin pool can be regulated at the transcription level. Neural and endocrine factors acting as secretory or proliferative stimuli can alter gap-junction coupling through many different regulatory pathways.

Connexins are present in the thymus and in cultured thymic epithelium from several species (Alves et al., 1994, 1995), however, the presence of connexins does not demonstrate the existence of functional gap junctions. Earlier studies with procion yellow suggested the presence of a functional syncytium (Kendall, 1985, 1986) and intercellular communication by dye coupling was characterized for cultured mouse and human thymic epithelium (Alves et al., 1994, 1995). Using cultured rat thymic epithelium, we have previously shown that the extent of cell coupling is modulated by factors known to affect the secretory activity of the cells (Head et al., 1996a, 1996b; Head et al., 1997). In this study, we extend the work to examine the effect of selected neuropeptides using the same cell-model system.

The cultured rat thymic cells form a stable cell type that is >98% epithelial after three to four subcultures (Kurz et al., 1996). The cultured cells share several similarities (expression of cortical and medullary markers, secretion of thymulin, and positive reaction to antibodies against calcitonin gene-related protein, CGRP) with the epithelium of the subcapsular cortex and a subpopulation of medullary epithelium. The subcapsular epithelium (type 1 cells; Wijngaert et al., 1984) completely surrounds the cortex and separates it from the connective tissues outside the thymus. The connective tissues contain small nonmyelinated nerves (Kendall et al., submitted) that form a lattice over the whole surface of the thymus. These fibers contain noradrenaline (NA), CGRP, neuropeptide Y (NPY), vasoactive intestinal peptide (VIP), and substance P (SP) (Felten et al., 1985; Felten and Felten, 1989; Weihe et al., 1989; Al-Shawaf, et al., 1991; Kendall and Al-Shawaf, 1991; Kendall et al., 1994; Kurz et al., 1995). Some of the nerves are also acetylcholinesterase positive (Al-Shawaf et al., 1991). Very few of the nerves accompany blood vessels, but some are close to mast cells (Weihe et al., 1989; Müller and Weihe, 1991) that are found in the connective tissue of the septa and capsule. The closest targets for the action of these neuropeptides and neurotransmitters would be the type 1 epithelial cells, or mast cells. Thus, neural action on epithelial cells could contribute to the control of the microenvironment in which early prothymocytes reside.

### RESULTS

### Dye Coupling (Figures 1 to 3)

The average CI of cells incubated in control medium was $0.85 \pm 0.12$ ($n = 118$) and more than 40% of cells were dye-coupled. Cells of all morphologies showed dye coupling. Transfer of dextran-conjugated dye occurred between some cells of syncitia with three or more constituent cells, as previously reported (Head et al., 1997). The CI was highly significantly reduced ($P < 0.0001$) both by $\gamma$-aminobutyric acid (GABA) (CI = $0.12 \pm 0.08$, $n = 52$) and NPY (CI = $0.13 \pm 0.09$, $n = 45$). A significant decrease ($P < 0.001$) was

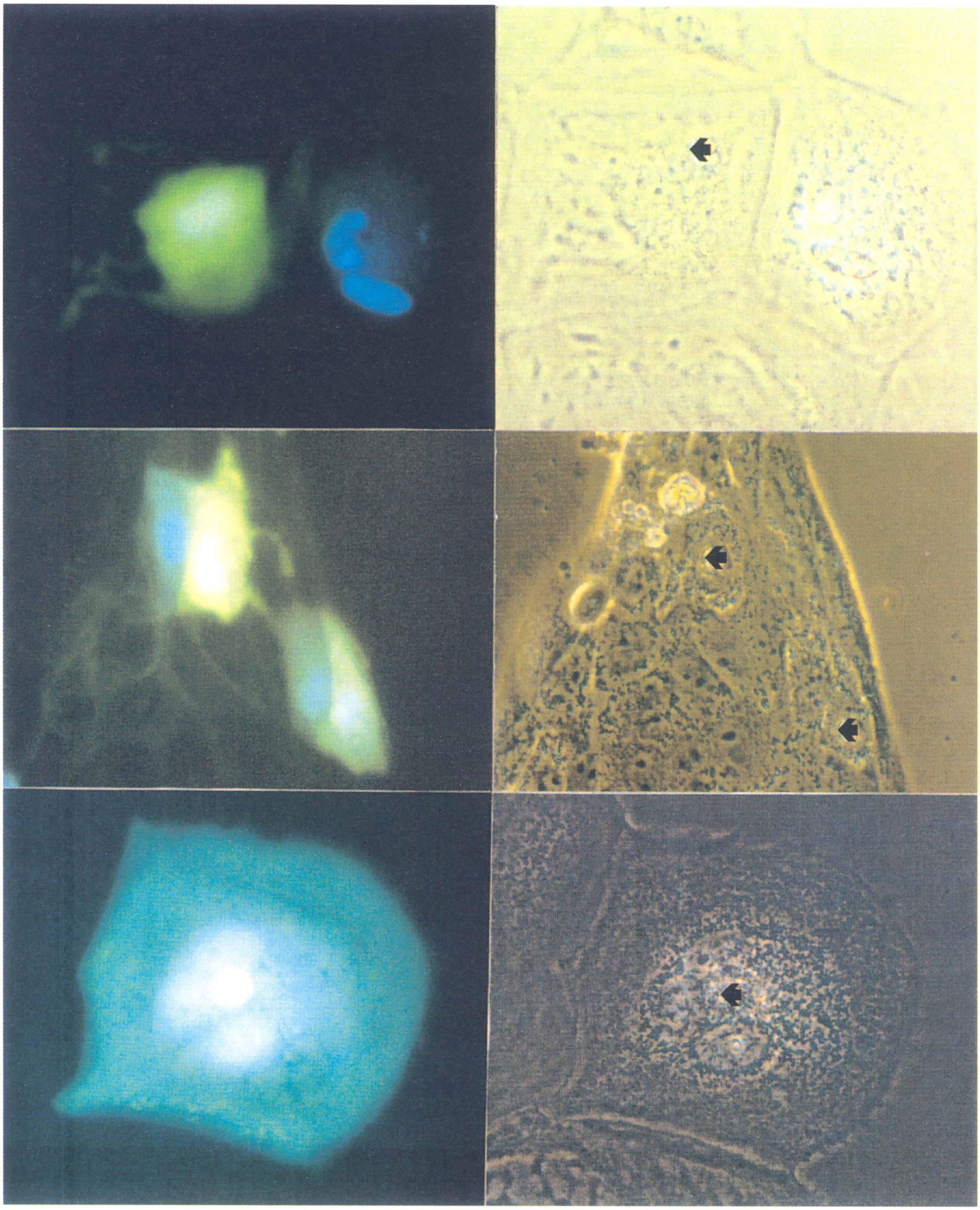

FIGURE 1 Cultured cells injected with LYD and CB (left) and paired phase contrast photos showing the injection site (right). Cells incubated in defined medium alone (top, ×450), 5-HT (middle, ×225), and SP (bottom, ×450). Several multinucleate cells are present (top and bottom photos). (See color plate VII)

produced by VIP (CI = 0.26 ± 0.11, $n$ = 47), and CGRP (CI = 0.26 ± 0.13, $n$ = 31), whereas SP (CI = 0.37 ± 0.14, $n$ = 38) and histamine (CI = 0.34 ± 0.16, $n$ = 32) had a lesser, but still significant effect ($P$ <

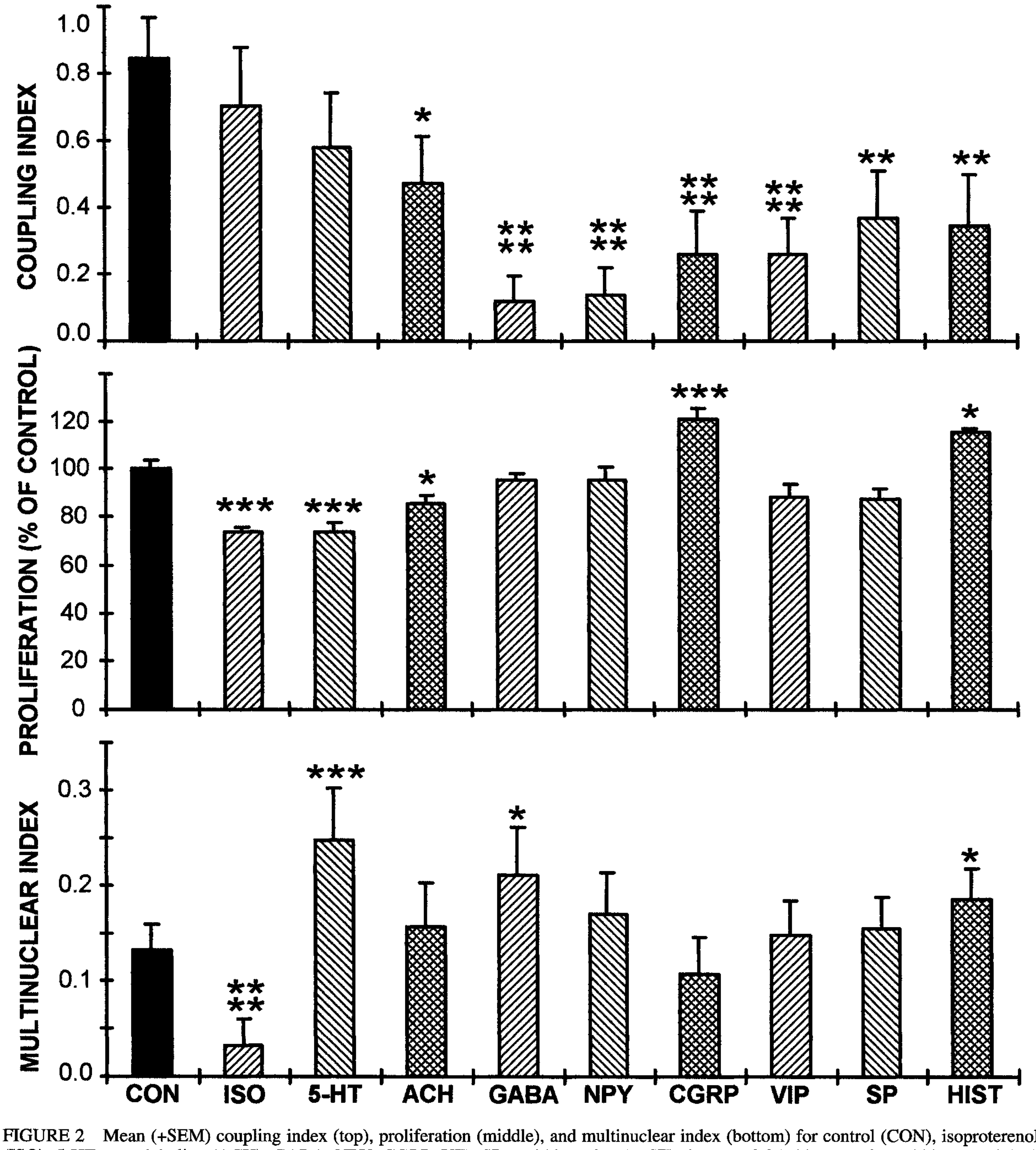

FIGURE 2 Mean (+SEM) coupling index (top), proliferation (middle), and multinuclear index (bottom) for control (CON), isoproterenol (ISO), 5-HT, acetylcholine (ACH), GABA, NPY, CGRP, VIP, SP, and histamine (HIST). * = $P < 0.05$; ** = $P < 0.01$; *** = $P < 0.005$; **** = $P < 0.0005$.

0.01). Acetylcholine ($n$ = 46), 5-hydroxytryptamine (5-HT) ($n$ = 31), and isoproterenol ($n$ = 37) did not significantly alter the average CI.

### Multinucleate Cells (Figure 2)

Multinucleate cells occurred in the control cultures (9.1%) with an MI (additional nuclei per cell) of 0.13 ± 0.03 ($n$ = 573; maximum number of nuclei in one cell is 4). Both the occurrence (2.5%) and the MI (0.03 ± 0.03, $P < 0.00002$, $n$ = 281) were reduced after incubation with isoproterenol. All other compounds either did not effect or caused an increase in the percentage of multinucleate cells. The increase in MI was only significant for 5-HT (MI = 0.25 ± 0.05, $P <$ 0.004, $n$ = 174; occurrence 21.3%), GABA (MI – 0.21 ± 0.05, $P < 0.03$, $n$ = 240; occurrence 15%), and histamine (MI – 0.17 ± 0.03, $P < 0.03$, $n$ = 457; occurrence 15.1%).

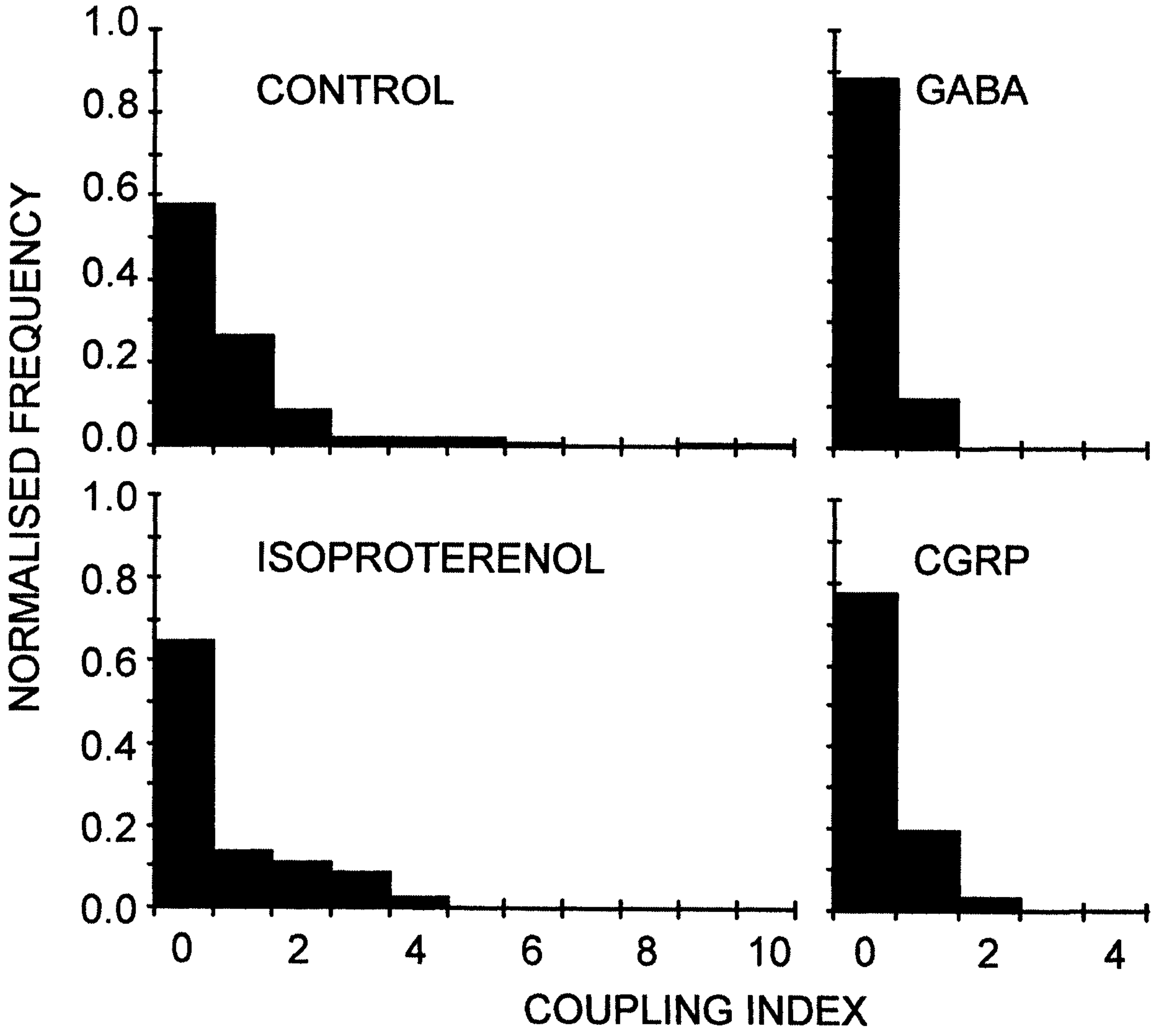

FIGURE 3 Normalized frequency histogram of CI for cells incubated with defined medium alone (control, top left), GABA (top right), isoproterenol (bottom left), and CGRP (bottom right).

### Proliferation (Figure 2)

Compared to controls, proliferation was highly significantly reduced by isoproterenol (74 ± 2%; $P$ = 0.002) and 5-HT (74 ± 3%; $P$ = 0.003), and significantly reduced by acetylcholine (86 ± 4%; $P$ < 0.05). Proliferation was highly significantly increased by CGRP (121 ± 4%; $P$ = 0.004) and significantly increased by histamine (115 ± 2%; $P$ < 0.02). Other compounds tested had no effect.

Since thymidine incorporation assesses nuclear not cellular proliferation, some consideration of the change in numbers of nuclei per cell is necessary. The decrease in numbers of nuclei observed with isoproterenol means that the decrease in cellular proliferation is less severe than that deduced from the observed decrease in nuclear proliferation. In the case of histamine, the converse is true; for 5-HT, an increased MI and decreased thymidine incorporation imply that cellular proliferation is even lower than the observed nuclear proliferation.

### Thymulin Determination (Figure 4)

Changes in supernatant thymulin levels were not seen after stimulation with GABA, NPY, CGRP, SP, or VIP. Compared to the previous control period, there were decreases in thymulin levels when cells were challenged with 5-HT (73 ± 3%, $P$ = 0.008), isoproterenol (73 ± 9%, $P$ = 0.03), and acetylcholine (52 ± 7%, $P$ = 0.02). Histamine also decreased thymulin levels in all cases, (84 ± 6%) although the change was not significant.

### DISCUSSION

Some research groups have sought to establish the action of selected neuropeptides on lymphocyte

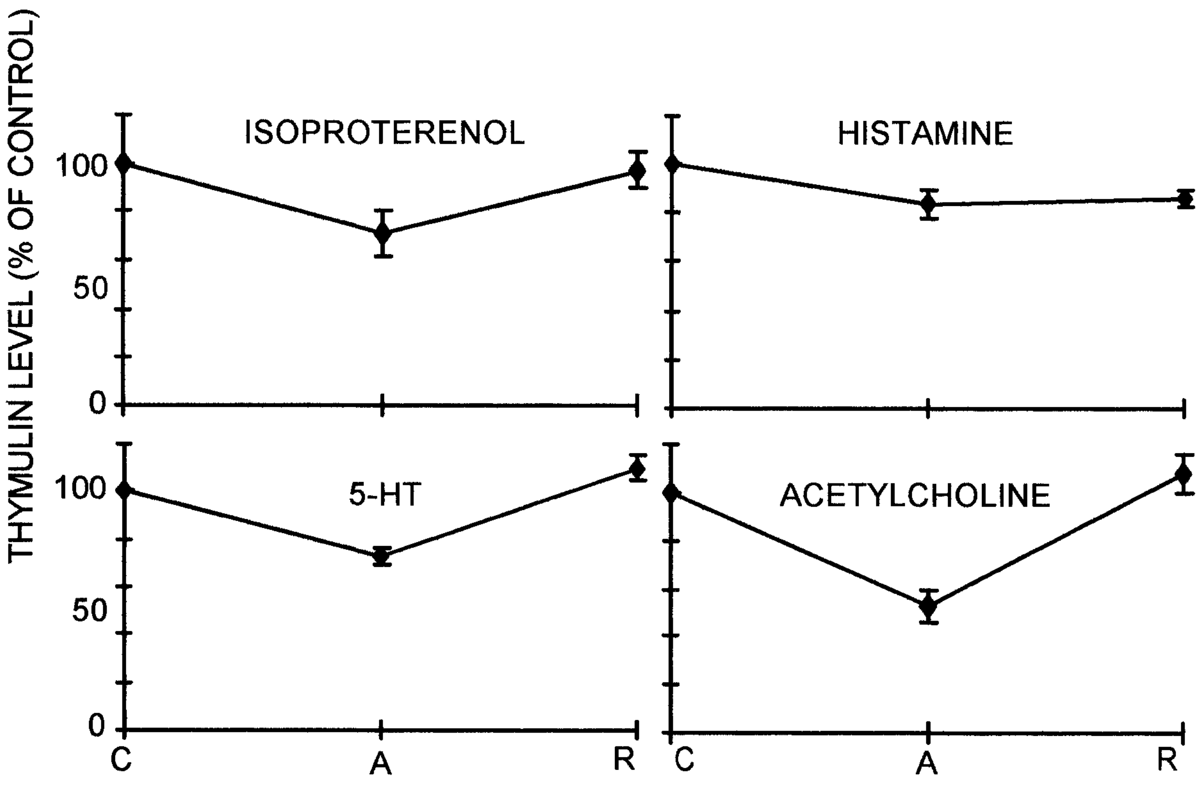

FIGURE 4 Percentage change and recovery (±SEM) of thymulin levels after incubation with isoproterenol (top left), histamine (top right), 5-HT (bottom left), and acetylcholine (bottom right). C = control; A = drug application; and R = recovery.

development by interactions with thymocytes (Vizi et al., 1995). Receptors for a wide variety of hormones and factors have been found on the cells of the microenvironment as well as on thymocytes, but little attention has been paid to direct actions of neuropeptides on thymic epithelial cells. The neuropeptides and neurotransmitters selected for study all occur in the subcapsular regions of the thymus (Felten et al., 1985; Felten and Felten, 1989; Weihe et al., 1989; Al-Shawaf, et al., 1991; Kendall and Al-Shawaf, 1991; Kendall et al., 1994; Kurz et al., 1995) and if released from these fibers would occur in relatively high concentrations around the subcapsular epithelial cells.

An appropriate experimental paradigm to test the hypothesis of direct neuropeptide or neurotransmitter action on thymic epithelium is provided by the cultured epithelial cells used here. These cells resemble type 1 (subcapsular/perivascular) and type 6 (subset of medullary cells) epithelium observed *in vivo*. Both of these types are unique in the thymus by having a highly secretory physiology with a measurable endocrine output. They have similar microtubular inclusions in man (Wijngaert et al., 1984), and in all species studied, react with the same CTES (clusters of thymic epithelial staining) group II antibodies (Kampinga et al., 1989). It has been suggested that the stem cells for thymic epithelium reside within these subsets (Lampert and Ritter; 1988).

Direct actions of neuropeptides and neurotransmitters on these cultured cells identify possible *in vivo* effects. However, our culture system cannot predict the effects of neuropeptides and neurotransmitters on other components of the thymic microenvironment or on their interactions with the epithelia.

The physiology of cultured thymic epithelial cells is complex, showing interconnected regulation of coupling, proliferation, and secretion (Head et al., 1997). Using a mixture of dyes, one of which does not pass through gap junctions, we have shown that factors known to positively influence the secretion of thymulin from thymic epithelial cells *in vivo* and *in vitro* decrease dye coupling in the DMEM/F12 medium used here. This change is probably mediated by increases in intracellular calcium (Buckingham et al., 1992). However, stimulation by neuropeptides does not show this correlation since those neuropeptides that decrease dye coupling had no effect on secretion. This suggests that neuropeptide-induced uncoupling does not involve calcium. Those neuropeptides that reduce thymulin levels had minimal or no effect on dye coupling (similar to the effects of thymulin itself; Head et al., 1997).

In other cells studied, increased gap-junction coupling generally decreases cellular proliferation (Trosko et al., 1993), although there are exceptions (e.g., C310T1/2 cells, where TGF$\beta$ causes an increase in both cell coupling and proliferation (Gibson et al., 1994). High levels of connexin 43 are found in rapidly proliferating lymphoid and hematopoietic centers (Rosendaal et al., 1994; Krenacs and Rosendaal, 1995). In our previous study of factors influencing secretion (Head et al., 1997), decreasing dye coupling inversely increased proliferation. In this study, this relationship is only true for CGRP and to a lesser extent, histamine. Thus, the action of the neuropeptides studied here is not primarily involved with thymulin secretion.

In the cultured thymic epithelial cells, there was a large decrease in CI and increased proliferation after GABA. In other systems such as the somatic musculature of *Ascaris lumbricoides* (Demello and Maldonado, 1985), GABA also causes gap-junction uncoupling. This effect on dye coupling could be enacted through lowered intracellular pH by GABA action at $GABA_A$ receptors (Takahashi and Copenhagen, 1996), as lowered pH decreases gap-junction permeability (Ueda et al., 1994).

Each component of the ensemble of intracellular signals (pH, calcium, cyclic nucleotides, kinases, etc.) has an effect on gap-junction communication. The interactions of these second messenger systems and the diversity of receptors from which they can be controlled make it difficult to predict the effects of a ligand on gap-junction function and turnover. For example, cAMP can regulate gap junction permeability, localization, or expression. In addition, it can interact with other second messenger cascades to activate complementary or antagonistic systems.

Typically, cAMP mediates the effects of isoproterenol through $\beta$-adrenergic activation of adenylate cyclase that usually leads to increased gap-junction coupling. In our cells, the average CI was not significantly higher although those syncytia present were larger. These cells are known to have $\beta$-adrenergic receptors (Kurz et al., 1997) that elevate intracellular cAMP and decrease proliferation, as observed in this study.

Neuropeptides may indirectly affect the secretory activity of endocrine tissue by altering signal transmission within the syncytium. Rises in intracellular calcium stimulating secretion in some organs do not start in all cells. They are initiated at one site and progress from cell to cell in a regular manner. The site of initiation can be different for different stimuli (e.g., vasopressin and noradrenaline in bile canaliculi; Combettes et al., 1994). Thus, disruption of gap-junction communication represses the recruitment of secretory cells and may change the dose-response characteristics of the syncytium. This has been shown for the response of human adrenal cells to corticotrophin (Munarisilem et al., 1995). Thus, neuropeptide action on thymic epithelial cells may directly initiate cellular responses or modulate the efficacy of other stimuli.

### MATERIALS AND METHODS

### Culture and Incubation with Stimulants

Thymic epithelial cell cultures were produced, characterized, and passaged according to the method of Kurz et al. (1996). Three culture media (some with additions from Sigma) were used in the study: DMEM/F12 (Gibco) alone; a defined medium, DMEM/F12, plus the following additions: 2 mM L-glutamine, 100 U/ml penicillin/streptomycin, and 25 $\mu$g/ml transferrin; and the standard culture medium comprising the defined medium plus 10% horse serum, 5 $\mu$g/ml insulin, 10 ng/ml cholera toxin, and epidermal growth factor (100 ng/ml). Stimulants (from Sigma) were added to the defined medium to the following final concentrations: acetylcholine, 1 mM; isoproterenol, 1 $\mu$M; GABA, 0.1 mM; NPY, 100 nM; CGRP, 100 nM; VIP, 100 nm; SP, 100 nM; 5-HT, 0.1 mM, and histamine, 0.1 mM.

### Dye Coupling

Cell cultures grown in the standard medium in 35-mm dishes were equilibrated for 2 hr in DMEM/F12 alone, and then incubated in a test medium for 24 to 36 hr. Cell cultures were transferred to a pressure microinjection apparatus (room temperature), and perfused with a modified Ringer's solution throughout the microinjection procedure, which was limited to a maximum of 15 min. Before injection, dye was applied to the external surface of the cells. If any incorporation of dye within the cells was observed, then no injections were performed at that site. Injection sites were widely spaced to prevent two injections into the same syncitium. Dye coupling was assessed by the injection of lucifer yellow dextran (LYD) at a 2.5 mM concentration (Molecular Probes; mol wt = 10,000) and Cascade blue (CB) at 5 mM concentration (Molecular Probes; mol wt = 644.77) close to or into the nucleus of individual cells. The dyes were visualized (blue fluorescence for CB and yellow fluorescence for LYD) by simultaneous excitation at 365 nm (dichroic filter, 395 nm; emission filter, 420nm) and the cells were observed by phase contrast microscopy.

To quantitate the extent of dye coupling for each culture, the results are expressed as a coupling index (CI), which is the average number of cells that are coupled to each injected cell. Thus, when is there is no coupling, CI = 0.

### Multinucleate Cells

The occurrence of multinucleate cells was estimated by counting the number of nuclei per cell in a sample of >150 cells, and expressed as a percentage of the population. Because multinucleate cells in these cultures have previously been observed to undergo mitosis without cytokinesis (Head et al., 1997), it is assumed here that each nucleus within a multinucleate cell contains a full complement of chromosomes. To represent the extent of chromosome duplication within these cells, the results are also expressed as the average number of additional nuclei per cell (the multinuclear index, or MI).

### Proliferation

Confluent cultures in 35-mm dishes were equilibrated, incubated for 24 hr with defined medium alone or with stimulants, 1 $\mu$Ci $^3$H-thymidine (Amersham, UK; Code TRK 565) was added to the test medium, and incorporation continued for 5 hr at 37°C. The supernatant was discarded and the cells washed sequentially with phosphate-buffered saline (PBS), water, methanol twice, 10% trichloracetic acid, and water (5 min each). The cells were dissolved in 1 ml of 0.3 M sodium hydroxide (15 min), neutralized with 1 ml of 0.3 M HCl, and then 2 ml of sample was added to 10 ml scintillation fluid (Hydroluma, Baker Chemicals, Code 8584) and the beta activity recorded. All experimental replicates had paired control cultures from passages 20 to 30, and changes in proliferation were calculated relative to these. Each condition was replicated four times.

### Estimation of Thymulin in Supernatants

Cells cultured as for the earlier dye-coupling experiments were equilibrated for 1 hr in DMEM/F12 alone. They were then incubated for 2 hr each in the defined medium, defined medium plus stimulant (at the concentrations previously listed), and again in the defined medium. Each experiment was replicated three times except isoproterenol (five times). The supernatant from each incubation was reserved and stored at −70°C before estimation of thymulin content by ELISA, as described in Head et al. (1997). Briefly, thymulin in supernatants was mixed with a polyclonal anti-thymulin antiserum (MK-R4) in a low-binding plate, and then transferred into a high-binding plate previously coated with synthetic thymulin. Only the free antiserum can bind to the thymulin coating, and this complex is visualized with a chromogen and the resultant color read in a plate reader at the appropriate wavelength. The lower the color, the higher the thymulin content in the sample tested.

### Statistics

Data are expressed as mean ± standard error of the mean and significance analyzed using Student's *t* tests.

### *Acknowledgements*

GMH was supported by the Medical Research Council, and JEGD by the Royal Society, with additional support provided by the University of London Central Research Fund and Smith Kline (1982) Foundation. RM, BvmP, and MDK were funded by the Volkswagen Stiftung, and MDK would like to thank the Welton Foundation for additional financial assistance.

### *References*

Al-Shawaf A., Cowen T., and Kendall M.D. (1991). Identification of neural profiles containing vasoactive intestinal polypeptide, acetylcholinesterase and catecholamines in the rat thymus. J. Anat. **174**: 131-143.

Alves L.A., Compos de Carvalho A.C., Lima E.O.C., Souza C.M.R.e, Dardenne M., Spray D.C., and Savino W. (1995). Functional gap junctions in thymic epithelial cells are formed by connexin 43. Eur. J. Immunol. **25**: 431-437.

Alves L.A., Compos de Carvalho A.C., Parreira-Martins L., Dardenne M., and Savino W. (1994). Intrathymic gap junction-mediated communication. In In vivo Immunology, Heinen E., Defresne M.P., Boniver J., and Geenen V., Eds. (New York: Plenum).

Buckingham J.C., Safieh B., Singh S., Arduino L.A., Cover P.O., and Kendall M.D. (1992). Interactions between the hypothalamo-pituitary adrenal axis and the thymus in the rat: A role for corticotrophin in the control of thymulin release. J. Neuroendocrin. **4**: 295-301.

Combettes L., Tran D., Tordjmann T., Laurent M., Berthon B., and Claret M. (1994). Ca2+-mobilizing hormones induce sequentially ordered Ca2+ signals in multicellular systems of rat hepatocytes. Biochem. J. **304**: 585-594.

Demello W.C., and Maldonado H. (1985) Synaptic inhibition and cell communication—impairment of cell-to-cell coupling produced by gamma-aminobutyric acid (GABA) in the somatic musculature of *Ascaris lumbricoides*. Cell Biol. Int. Rep. **9**: 803-813.

Felten D.L., and Felten S.Y. (1989). Innervation of the thymus. Thymus Update **2**: 73-88.

Felten D.L., Felten S.Y., Carlson S.L., Olshowka J.A., and Livnat S. (1985). Noradrenergic and peptidergic innervation in lymphoid tissue. J. Immunol. **135**: 7555-7655.

Gibson D.F., Hossain M.Z., Goldberg G.S., Acevedo P., and Bertram J.S. (1994). The mitogenic effects of transforming growth factor beta 1 and factor beta 2 in C3H10T1/2 cells occur in the presence of enhanced gap junctional communication. Cell Growth Diff. **5**: 687-696.

Head G.M., Kranz A., and Kendall M.D. (1996a). The dye coupling of rat thymic epithelial cells in culture. J. Anat. **189**: 227-228.

Head G.M., Kranz A., Kendall M.D., and Downing J.E.G. (1996b). Proliferation and dye coupling in cultured rat thymic epithelial cells. J. Physiol. **493P**: 63P-64P.

Head G.M., Mentlein R., Kranz A., Downing J.E.G., and Kendall M.D. (1997). Modulation of dye-coupling in cultured rat thymic epithelium by factors involved in thymulin secretion. J. Anat. **191**: 355-365.

Lampert I.A., and Ritter M.A. (1988). The origin of the diverse epithelial cells of the thymus: Is there a common stem cell? Thymus Update **1**: 5-25.

Kampinga J., Berges S., Boyd R.L., Brekelmans P., Colic M., van Ewijk W., Kendall M.D., Ladyman H., Nieuwenhuis P., Ritter M.A., Schuurman H-J., and Tournefier A. (1989). Thymic epithelial antibodies: Immunohistological analysis and introduction of nomenclature. Summary of the Epithelium Workshop held at the 2nd Workshop, "The Thymus. Histophysiology and Dynamics in the Immune System." Thymus **13**: 165-173.

Kendall M.D. (1985). The outer and inner thymus cortex is a functional syncytium. Cell Biol. Int. Rep. **9**: 3.

Kendall M.D. (1986). The syncytial nature of epithelial cells in the thymic cortex. J. Anat. **147**: 95-106.

Kendall M.D., Al-Asam H., and Zaidi S.A.A. (Submitted). The morphology of large nerves in the rat thymus.

Kendall M.D., and Al-Shawaf A.A. (1991). Current knowledge on the innervation of the rat thymus gland. Brain Behav. Immun. **5**: 9-28.

Kendall M.D., Atkinson B.A., Munoz F.J., Riva C. de la, and Clarke A.G. (1994). The noradrenergic innervation of the rat thymus during pregnancy and in the post partum period. J. Anat. **185**: 617-625.

Krenacs T., and Rosendaal M. (1995). Immunohistological detection of gap-junctions in human lymphoid-tissue - connexin 43 in follicular dendritic and lymphoendothelial cells. J. Histochem. Cytochem. **43**: 1125-1137.

Kumar N.M., and Gilula N.B. (1992). Molecular biology and genetics of gap junction channels. Sem. Cell Biol. **3**: 3-16.

Kurz B., Gaudecker B. von, Kranz A., Krisch B., and Mentlein R. (1995). Calcitonin gene-related peptide and its receptor in the thymus. Peptides **16**: 1497-1503.

Kurz B., Gaudecker B. von, Krisch G., and Mentlein R. (1996). Rat epithelial cells in vitro and in situ: Characterization by immunocytochemistry and morphology. Cell Tiss. Res. **283**: 221-229.

Kurz B., Feindt J., Gaudecker B. von, Kranz A., Loppnow H., and Mentlein R. (1997). $\beta$-adrenoceptor-mediated effects in rat cultured thymic epithelial cells. Brit. J. Pharmacol. **120**: 1401-1408.

Loewenstein W.R. (1979). Junctional intercellular communication and the control of growth. Biochim. Biophys. Acta **560**: 1-65.

Meda P., Bosco D., Giordano E., and Chanson M. (1991). Junctional coupling modulation by secretagogues in two cell pancreatic systems. In Biophysics of Gap Junction Channels, Peracchia, C., Ed. (Boca Raton, FL: CRC Press), pp. 191-208.

Müller S., and Weihe E. (1991). Interrelation of peptidergic innervation with mast cells and ED-1 positive cells in the rat thymus. Brain Behav. Immun. **5**: 55-72.

Munarisilem Y., Lebrethon M.C., Morand I., Rousset B., and Saez J.M. (1995). Gap junction-mediated cell-to-cell communication in bovine and human adrenal-cells: A process whereby cells increase their responsiveness to physiological corticotrophin concentrations. J. Clin. Invest. **95**: 1429-1439.

Ngezhayo A., and Kolb H. (1993). Gap junctional conductance tunes phase difference of cholecystokinin evoked calcium oscillations in pairs of pancreatic acinar cells. Pflug. Arch. E. J. Physiol. **422**: 413-415.

Pandol S.J. (1994). Pancreatic acinar cell signalling mechanisms. Curr. Op. Gasteroent. **10**: 485-490.

Rosendaal M., Green C.R., Rahman A., and Morgan D. (1994). Upregulation of the connexin-43(+) gap junction network in hematopoietic-tissue before the growth of stem cells. J. Cell Sci. **107**: 29-37.

Santos-Sacchi J. (1991). Isolated supporting cells from the organ of Corti: Some whole cell electrical characteristics and estimates of gap junctional conductance. Hearing Res. **52**: 89-98.

Takahashi K.I., and Copenhagen D.R. (1996). Modulation of neuronal function by intracellular pH. Neurosci. Res. **24**: 109-116.

Trosko J.E., Madhukar B.V., and Chang C.C. (1993). Endogenous and exogenous modulation of gap junctional intercellular communication: Toxicological and pharmacological implications. Life Sci. **53**: 1-19.

Ueda F., Kameda Y., Yamamoto Y., and Shibata Y. (1994). Beta-adrenergic regulation of gap junctional intercellular communication in cultured rabbit gastric epithelial cells. J. Pharmacol. Exp. Therap. **271**: 397-402.

Vizi E.S., Ordo E., Osipenko O.N., Hasko G., and Elenkov I.J. (1995). Neurochemical, electrophysiological and immunocytochemical evidence for a noradrenergic link between the sympathetic nervous system and thymocytes. Neurosci. **68**: 1263-1276.

Weihe E., Müller S., Fink T., Zentel H.J. (1989). Tachykinins, calcitonin gene-related peptide and neuropeptide Y in nerves of the mammalian thymus: Interactions with mast cells in autonomic and sensory neuroimmunomodulation? Neurosci. Lett. **100**: 77-82.

Wijngaert F.P. van de, Kendall M.D., Schuurman H.-J., Rademakers L.H.M.P., and Kater L. (1984). Heterogeneity of human thymus epithelial cells at the ultrastructural level. Cell. Tiss. Res. **237**: 227-237.

Yeager M., and Nicholson B.J. (1996). Structure of gap junction intracellular channels. Curr. Op. Struct. Biol. **6**: 183-192.

